# *CDC25C* as a Predictive Biomarker for Immune Checkpoint Inhibitors in Patients With Lung Adenocarcinoma

**DOI:** 10.3389/fonc.2022.867788

**Published:** 2022-04-29

**Authors:** Wengang Zhang, Xiaoling Shang, Fei Yang, Wenfei Han, Handai Xia, Ni Liu, Yanguo Liu, Xiuwen Wang

**Affiliations:** Department of Medical Oncology, Qilu Hospital, Cheeloo College of Medicine, Shandong University, Jinan, China

**Keywords:** *CDC25C*, immune checkpoint inhibitors, biomarker, lung adenocarcinoma, tumor microenvironment

## Abstract

The application of immune checkpoint inhibitors (ICIs) in non-small cell lung cancer has significantly improved patient survival. However, most patients fail to respond to ICIs or develop drug resistance during treatment. Therefore, novel biomarkers are needed to predict the efficacy of ICIs or provide clues on how to overcome drug resistance. Here, it was revealed that cell division cycle 25C (*CDC25C*) expression was upregulated in lung adenocarcinoma (LUAD) compared to that of normal lung tissue in multiple databases. This was further verified by q-PCR. Furthermore, higher *CDC25C* expression was associated with shorter overall survival and worse pathological stage. Most importantly, a higher *CDC25C* expression was associated with shorter progression-free survival in LUAD patients treated with nivolumab, suggesting the role of the cell cycle in immunotherapy. In addition, *CDC25C* expression was significantly associated with immune cell infiltration and immune-related signatures in the LUAD tumor microenvironment. Moreover, *CDC25C* was differentially expressed and correlated with overall survival in multiple tumors, indicating that *CDC25C* is a broad-spectrum biomarker. Taken together, our study demonstrates that *CDC25C* is a prognostic biomarker for LUAD patients, especially for patients treated with ICIs. Our study also provides strong evidence for the role of the cell cycle in ICIs therapy and tumor microenvironment.

## Introduction

Lung cancer is one of the most common cancers in the world. According to the GLOBOCAN 2020 estimation, there were approximately 19.3 million newly diagnosed cancer patients in 2020, of which lung cancer accounted for 11.4%. Notably, the latest data show that lung cancer ranks first in cancer-related deaths (1, 2). Based on the pathological classification, lung cancer can be divided into small cell lung cancer and non-small cell lung cancer (NSCLC), in which lung adenocarcinoma (LUAD) is the most common subtype (3). Over the past few decades, advances have been made in the treatment of NSCLC, including targeted therapy and immune checkpoint inhibitors (ICIs) therapy, especially for patients with LUAD (4). The introduction of ICIs, including antibodies targeting programmed cell death protein 1 (PD-1), programmed death-ligand 1 (PD-L1), and cytotoxic T lymphocyte antigen 4 (CTLA-4), have significantly prolonged survival of patients with advanced NSCLC, with 5-year survival rates from < 5% to 23.2%, reaching 31.9% in patients with PD-L1 >50% (5–7). However, only a small portion of patients (approximately 14% - 40%) with NSCLC responded to ICIs, even in patients with high PD-L1 expression (8–11). Therefore, developing novel biomarkers that predict the response to ICIs and screen patients who benefit from ICIs-based therapies is a crucial mandate. Currently, it has been demonstrated that patients with high PD-L1 expression and high tumor mutation burden (TMB) are more likely to benefit from ICIs (12). Nevertheless, clinicians cannot accurately identify patients that would benefit from ICIs treatment since some patients with low PD-L1 or TMB were reported to respond to ICIs (13, 14). Indeed, various factors involved in the tumor microenvironment (TME) or cancer-immunity cycle participate in the regulation of ICIs therapy. Notably, recent studies have found that many genes play critical roles in the transformation of TME types. Guo et al. revealed that *Zeb1* induced an immunosuppressive TME (15), which mediated tumor cell escaping from immune surveillance. Therefore, it is necessary to explore more biomarkers that influence or predict the efficacy of ICIs from this perspective.

Aberrant cell cycle is one of the crucial mechanisms leading to uncontrolled proliferation of cancer cells, which is an important hallmark of cancer (16). Currently, strong evidence indicates cell cycle progression is closely related to cancer invasion, metastasis, anti-apoptosis, and chemotherapy-resistance (17). Therefore, targeting cell-cycle proteins is a promising anti-tumor modality and has been extensively studied (16). Notably, cyclin-dependent kinase 4/6 (*CDK4/6*, cell cycle-related genes) inhibitors have shown significant activity against several cancers and some (namely palbociclib, ribociclib and abemaciclib) are approved for treatment of patients with advanced breast cancer (18). Actually, the regulation of cell cycle involves numerous genes. Besides *CDK4/6*, the cell division cyclin 25 (*CDC25*) family, consisting of *CDC25A*, *CDC25B*, and *CDC25C*, are also involved cell-cycle progression (19). Among them, *CDC25C*, a specific cyclin and dual-specificity phosphatase, plays a predominant role in regulating the initiation of cell division and controlling the cell cycle (19). Currently, *CDC25C* has been found to play a pro-tumor role in various tumors. Higher expression of *CDC25C* has been observed in multiple cancers, including bladder (20), gastric (21) and colorectal cancers (22), associated with poor prognosis. Intriguingly, reciprocal activation between *CDC25C* and *cyclin B1/CDK1* is observed. In other words, *CDC25C* activates *cyclin B1/CDK1* complex, which in turn phosphorylates *CDC25C*, thereby accelerating cell division by positive feedback (19). Notably, accumulating evidence suggested that inhibition of *CDKs* not only contributes to cell cycle arrest but also triggers anti-tumor immunity (23–26) and enhances the efficiency of anti-PD-1 therapy (27), thus *CDC25C* might also have potential immunoregulatory effect *via* interaction with *CDKs*. However, the role of *CDC25C* in lung cancer especially its influence on the tumor immune microenvironment and ICIs efficacies has not been clarified.

In the study, the prognostic role of *CDC25C* in patients with LUAD was investigated, especially in patients treated with ICIs, using the TCGA and GEO databases. In addition, the infiltration levels of various immune cells or immune-related signatures in TME and gene enrichment analysis were evaluated to explore the potential mechanism of *CDC25C* in ICIs treatment. Finally, the role of *CDC25C* in pan-cancer was analyzed using TCGA database.

## Materials and Methods

### RNA Seq and Clinical Data From TCGA and GEO

RNA-seq and clinical data were downloaded from The Cancer Genome Atlas (TCGA) for 33 cancers, including LUAD and lung squamous carcinoma (LUSC). LUAD data consisted of 59 adjacent nontumor tissues and 535 LUAD tissues. LUSC data consisted of 49 adjacent nontumor tissues and 502 LUSC tissues. In addition, GSE13213, GSE157001, GSE157009, and GSE126044 datasets were downloaded from the GEO database. Patients were classified as responders when the response (PR) or stable disease (SD) is > 6 months according to Response Evaluation Criteria in Solid Tumors (RECIST) ver. 1.1. When progressive disease (PD) or SD is ≤ 6 months, patients were classified as non-responders by RECIST ver. 1.1.

### *CDC25C* Expression Analysis in LUAD and LUSC

The *CDC25C* expression in LUAD and LUSC was analyzed by the R software ggplot2 package using the downloaded TCGA data. The Oncomine database (28) was then used to validate the expression of *CDC25C* in LUAD. Next, the Human Protein Atlas (HPA) database was used to explore the expression of *CDC25C* in LUAD versus normal lung tissue at the protein level. In addition, q-PCR was performed using lung normal epithelial cell line (BEAS-2B) and LUAD cell lines (PC-9 and H1299) for further validation at the mRNA level. Based on clinical data from TCGA LUAD, *CDC25C* expression was analyzed in patients stratified by age, gender, smoking history, T-stage, N-stage, M-stage, and pathological stage. Among the pathological stage analysis, we defined stage I and II as local, stage III as locally advanced, and stage IV as metastatic. Furthermore, through the UALCAN database (29), the relationship between *CDC25C* expression and *TP53* mutation status was explored in LUAD patients.

### mRNA-Based Survival Analysis of LUAD and LUSC Patients

Survival analysis was performed using LUAD and LUSC data from TCGA, including RNA-seq and survival data. The cut-off value for high and low *CDC25C* in this study was determined by the median *CDC25C* within each cancer type. According to the median risk score, the patients were divided into low- and high-risk groups. The formula is as follows: risk score = Exp × Coef (Exp = gene expression value, Coef = coefficient). The “survminer” and “survivor” packages were used for the analyses. The ROC curves were constructed in LUAD using “pROC” and “tmieROC” packages. Univariate and multivariate Cox regression analyses were performed to further investigate the correlation between multivariable characteristics and overall survival (OS) using LUAD data (30). The GEO datasets were used to further validate the relationship between *CDC25C* expression and lung cancer prognosis.

### Pathway Enrichment Analysis

The gene expression profiles of LUAD from TCGA were downloaded and partitioned into two groups according to the median value of *CDC25C* expression. The R package “edgeR” was used to obtain the differentially expressed genes between the two groups (31). In addition, Gene Set Enrichment Analysis (GSEA) was performed using the “ClusterProfiler” package (32). A gene set with a p-value lower than 0.05 was considered significantly enriched in Kyoto Encyclopedia of Genes and Genomes (KEGG) and Gene Ontology (GO) terms.

### Immune Cell Infiltration Analysis in LUAD

As described in previous literature, the single-sample gene set enrichment analysis (ssGSEA) was performed to quantify the relative abundance of tumor-infiltrating immune cells in LUAD patients with a high or low *CDC25C* expression using the “GSVA” R package (33, 34). Then, Spearman was used to analyze the correlation between immune cell infiltration and *CDC25C* expression. Next, the relationship between *CDC25C* and multiple types of immune cell infiltration was analyzed in LUAD tissues using TISIDB database (35). In addition, the TIMER database was used to explore the relationship between the copy number of *CDC25C* and immune cell infiltration. Moreover, four immune-related signatures (immuno-stimulators, immuno-inhibitors, MHC molecules, and chemokines) were compared between high *CDC25C* and low *CDC25C* expression LUAD using data from the TCGA database.

### Identification of 10 Hub Genes Co-Expressed With *CDC25C*

Through the STRING database (36), we found 10 hub genes that were co-expressed with *CDC25C* in LUAD patients. Then, GEPIA (http://gepia.cancer-pku.cn/index.html) was used to explore the correlation between *CDC25C* expression and these hub genes. In addition, the expression of these hub genes in normal and LUAD tissues was explored using TCGA database. Furthermore, the prognostic values of the 10 hub genes were explored using TCGA LUAD data.

### Analysis of *CDC25C* in Pan-Cancer

The expression of *CDC25C* in pan-cancer was analyzed using the UALCAN database (29) and the Oncomine database (28). Then, through the TCGA database, the relationship between *CDC25C* expression and OS was analyzed in pan-cancer.

### RNA Extraction and q-PCR

Total RNA of BEAS-2B, PC9, and H1299 cells was isolated using the TRIzol reagent (Thermo Fisher Scientific). Then, cDNA was synthesized from 1 ug of total RNA using reverse transcription reagents (Accurate Biology, China) according to the manufacturer’s protocol. Next, LightCycler 480 device (Roche Diagnostics) and SYBR^®^ Green Premix Pro Taq HS qPCR kit (Accurate Biology, China) were used to perform PCR. β-actin was used to normalize gene expression. Primer sequences were as follows:

*CDC25C* forward primer: GCAGAAGTGGCCTATATCGCT

*CDC25C* reverse primer: TTCCACCTGCTTCAGTCTTGG

*β-actin* forward primer: GAAGAGCTACGAGCTGCCTGA

*β-actin* reverse primer: CAGACAGCACTGTGTTGGCG

### Statistical Analysis

The *CDC25C* expression between tumor and normal tissues was analyzed by Wilcoxon rank-sum test, and the q-PCR results were analyzed by t-test. In addition, the correlation between *CDC25C* and clinical characteristic variables was analyzed by the Pearson chi-square test. The Kaplan-Meier method and log-rank tests were used to perform the survival analysis. Moreover, univariate and multivariate analyses were conducted using the Cox proportional hazards regression model. In this study, the R software (version 4.1.1) and the GraphPad Prism (version 9.0) were used for data analysis. Also, multiple online databases, including TIMER, UALCAN, HPA, GEPIA, and STRING, were used for analysis. A two-tailed P-value < 0.05 was considered statistically significant.

## Results

### The Expression Level of *CDC25C* in Lung Cancer

**Figure 1:**
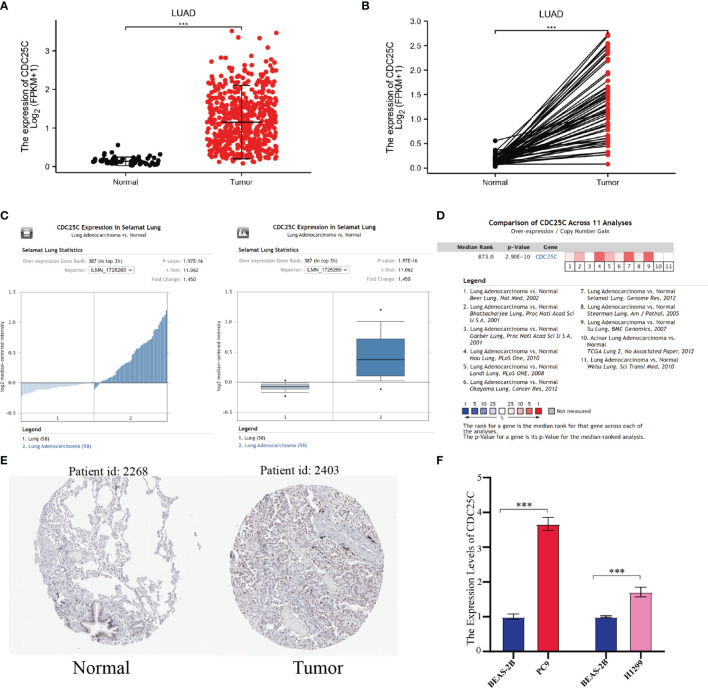
The expression of *CDC25C* in lung cancer. **(A, B)** *CDC25C* expression between tumors and normal tissues of non-paired **(A)** and paired **(B)** LUAD patients from the TCGA database. **(C)** By t-test, box plot and peak plot of *CDC25C* in LUAD. **(D)** A meta-analysis using data from 11 published studies showed that *CDC25C* was upregulated in LUAD. **(E)** Immunohistochemical results of *CDC25C* in normal lung tissues and LUAD tissues from the human protein atlas database. **(F)** q-PCR analysis for *CDC25C* in normal lung epithelial cell line (BEAS-2B) and lung adenocarcinoma cell lines (PC9, H1299) (***P < 0.001).

First, to investigate whether *CDC25C* was differentially expressed between lung cancer and normal tissue, LUAD and lung squamous carcinoma (LUSC) RNA-seq data were downloaded from TCGA. It was observed that *CDC25C* expression was significantly upregulated in both LUAD and LUSC compared with normal tissues (all P-values < 0.001) (**Figures 1A, B** and **Figures S1A, B** **)**. Although *CDC25C* expression was significantly upregulated in LUSC, subsequent survival analysis revealed no significant correlation between *CDC25C* expression and patient survival. Therefore, this study mainly focused on the relationship between *CDC25C* and LUAD. Next, the Oncomine database was used for further validation. By the t-test, box plot and peak plot of *CDC25C* in LUAD of Selamat Lung were shown in **Figure 1C** (t-test = 11.062, Fold Change = 1.45, P = 1.97E-16). A meta-analysis using data from 11 published studies showed that *CDC25C* was increased in LUAD compared to normal lung tissues (Median Rank = 873.0, P = 2.90E-10) (**Figure 1D**). Moreover, the HPA database was used to further validate this result at the protein level. As illustrated in **Figure 1E**, the expression of *CDC25C* was higher in LUAD tissues than in normal lung tissues. We further explored the differential expression of *CDC25C* by q-PCR using a normal lung epithelial cell line (BEAS-2B) and LUAD cell lines (PC9 and H1299). As shown in **Figure 1F**, the *CDC25C* expression was higher in PC9 (P < 0.001) and H1299 (P < 0.001) when compared to that of BEAS-2B.

### Prognostic Significance of *CDC25C* Expression in LUAD

**Figure 2:**
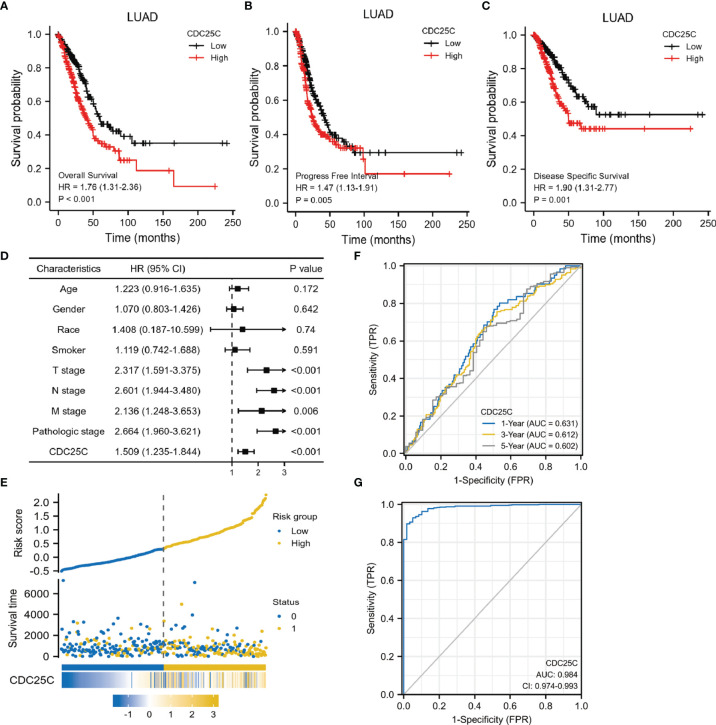
Prognostic role of *CDC25C* in lung cancer. **(A–C)** Correlation among OS, PFI, DSS and *CDC25C* expression of LUAD in TCGA database. **(D)** Forest plot of the univariate analysis results assessing independent prognostic factors for OS of LUAD. **(E)** The high and low risk maps. **(F)** The AUC curve of 1, 3 and 5 years to forecast the survival of LUAD patients. **(G)** The diagnostic value of *CDC25C* in LUAD patients.

Survival analysis was conducted using the TCGA database to determine the correlation between *CDC25C* expression and lung cancer prognosis. Results showed that higher *CDC25C* expression was statistically associated with a shorter overall survival (OS) (P < 0.001), shorter progress free interval (PFI) (P = 0.005) and shorter disease-specific survival (DSS) (P = 0.001) in LUAD (**Figures 2A** **–C**), but not in LUSC (all P values > 0.05) (**Figures S1C** **–E**). For further validation, survival analysis was performed using LUAD and LUSC data from the GEO database and results were strikingly similar (**Figures S1F** **–H**). In addition, an univariate analysis demonstrated that T-stage (HR: 2.317, 95% CI: 1.591-3.375, p < 0.001), N-stage (HR: 2.601, 95% CI: 1.944-3.480, p < 0.001), M-stage (HR: 2.136, 95%CI: 1.248-3.653, p = 0.006), pathological stage (HR: 2.664, 95%CI: 1.960-3.621, p **<** 0.001), and *CDC25C* expression (HR: 1.509, 95%CI: 1.235-1.844, p **<** 0.001) were independent prognostic biomarkers for LUAD patients (**Figure 2D**). Meanwhile, high *CDC25C* expression was found to be an independent prognosis biomarker for poor OS for LUAD patients in multivariate analysis (HR: 1.637, 95%CI: 1.267-2.116, p < 0.001) (**Table 1**). Based on the risk score, we divided the population into high and low-risk groups and found that the high-risk group had a worse prognosis and higher *CDC25C* expression, when compared to the low-risk group (**Figure 2E**). Of note, *CDC25C* could better predict the 1-year (AUC 0.631), 3-year (AUC 0.612) and 5-year (AUC 0.602) survival rate of LUAD patients (**Figure 2F**). The ROC curve presented that *CDC25C* had a very low false-positive rate and a very high true-positive rate (AUC:0.984, CI:0.974-0.993) (**Figure 2G**), which indicated that *CDC25C* possessed an incredible diagnostic power for LUAD.

**Table 1:** Correlation between overall survival and multivariable characteristics in TCGA patients *via* Cox regression multivariate survival model.

Characteristics	Total(N)	Univariate analysis		Multivariate analysis	
		Hazard ratio (95% CI)	P value	Hazard ratio (95% CI)	P value
Age	516				
<=65	255	Reference			
>65	261	1.223 (0.916-1.635)	0.172	1.289 (0.881-1.886)	0.191
Gender	526				
Female	280	Reference			
Male	246	1.070 (0.803-1.426)	0.642	0.995 (0.680-1.455)	0.978
Race	468				
Asian	7	Reference			
Black or African American	55	1.408 (0.187-10.599)	0.740	2.009 (0.244-16.547)	0.517
White	406	2.030 (0.284-14.519)	0.481	2.959 (0.401-21.840)	0.288
Smoker	512				
Yes	440	Reference			
No	72	1.119 (0.742-1.688)	0.591	0.910 (0.532-1.557)	0.730
T stage	523				
T1&T2	457	Reference			
T3&T4	66	2.317 (1.591-3.375)	**<0.001**	2.177 (1.254-3.777)	**0.006**
N stage	510				
N0	343	Reference			
N1&N2&N3	167	2.601 (1.944-3.480)	**<0.001**	2.287 (1.483-3.527)	**<0.001**
M stage	377				
M0	352	Reference			
M1	25	2.136 (1.248-3.653)	**0.006**	1.342 (0.644-2.793)	0.432
Pathologic stage	518				
Stage I&Stage II	411	Reference			
Stage III&Stage IV	107	2.664 (1.960-3.621)	**<0.001**	1.039 (0.603-1.790)	0.890
*CDC25C*	526	1.509 (1.235-1.844)	**<0.001**	1.637 (1.267-2.116)	**<0.001**

Bold values indicate P < 0.05.

### Relationship Between *CDC25C* Expression and Clinical Features in LUAD Patients

**Figure 3:**
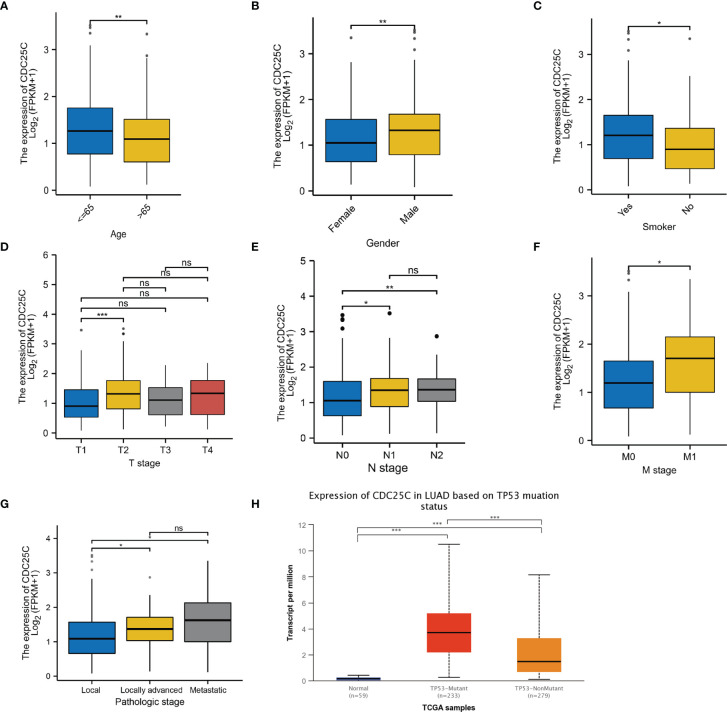
The relationship between *CDC25C* expression and clinical features of LUAD. **(A–G)** Analysis of LUAD data from the TCGA database showed that *CDC25C* expression correlated with age, gender, smoking history, T-stage, N-stage, M-stage, and pathological stage. **(H)** Through the UALCAN database, we found that *CDC25C* expression correlated with *TP53* mutation status in LUAD (ns, not significant, *P < 0.05, **P < 0.01, ***P < 0.001).

By grouping TCGA data according to different clinical characteristics, we observed that *CDC25C* expression was closely associated with several clinical characteristics of LUAD patients. LUAD patients of low age (P < 0.01), male (P < 0.01), and with a history of smoking (P < 0.05), expressed higher *CDC25C* (**Figures 3A** **–C**). Besides, *CDC25C* was more highly expressed in T2-stage tumors than in T1-stage ones (P < 0.001) (**Figure 3D**). Regarding N-stage, higher *CDC25C* expression was observed in N1 and N2 stage tumors than in N0 stage tumors (all P values < 0.05) (**Figure 3E**). Meanwhile, M1-stage tumors expressed higher *CDC25C* than the M0-stage (P < 0.05) (**Figure 3F**). In addition, higher *CDC25C* expression significantly correlated with later pathological stages (local vs locally advanced: P < 0.05; local vs metastatic: P < 0.05) (**Figure 3G**). Moreover, LUAD bearing *TP53* mutation had a higher *CDC25C* expression (P < 0.001) (**Figure 3H**). Thus, *CDC25C* expression was associated with the malignant progression of LUAD.

### Pathway Enrichment Analysis of *CDC25C* in LUAD

**Figure 4:**
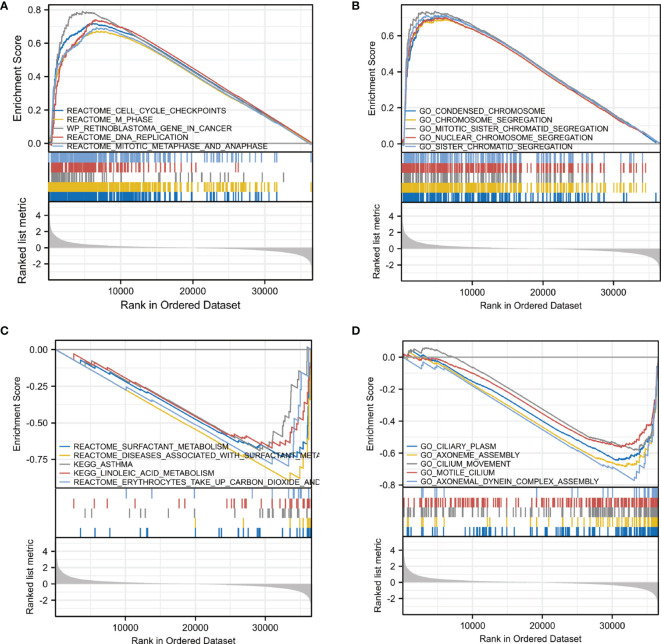
Pathway enrichment analysis of *CDC25C* in LUAD. **(A, B)** Gene enrichment plots displayed enrichment pathways in the high *CDC25C* expression LUAD. **(C, D)** Gene enrichment plots displayed enrichment pathways in the low *CDC25C* expression LUAD.

In order to elucidate the underlying mechanism of *CDC25C* action in LUAD, functional enrichment analysis was performed. First, we identified ten enriched signaling pathways positively associated with *CDC25C* expression (**Figures 4A, B** and **Table 2**), including: condensed chromosome, chromosome segregation, mitotic sister chromatid segregation, nuclear chromosome segregation, sister chromatid segregation, cell cycle checkpoints, M phase, DNA replication, mitotic metaphase, and anaphase. Then, ten enriched signaling pathways negatively associated with *CDC25C* expression were also identified (**Figures 4C, D**, and **Table 2**), including: axonemal dynein complex assembly, surfactant metabolism, diseases associated with surfactant metabolism and linoleic acid metabolism. From the enrichment analysis results, it was evident that *CDC25C* was associated with cell mitosis and metabolism.

**Table 2:** Signaling pathways most significantly correlated with *CDC25C* expression based on their normalized enrichment score (NES) and p-value.

	GO NAME	NES	p-value	FDR
Positive	condensed_chromosome	2.626	0.002	0.030
	chromosome_segregation	2.625	0.002	0.030
	mitotic_sister_chromatid_segregation	2.620	0.002	0.030
	nuclear_chromosome_segregation	2.618	0.002	0.030
	sister_chromatid_segregation	2.607	0.002	0.030
Negative	ciliary_plasm	-2.344	0.002	0.034
	axoneme_assembly	-2.268	0.002	0.032
	cilium_movement	-2.197	0.003	0.035
	motile_cilium	-2.190	0.003	0.038
	axonemal_dynein_complex_assembly	-2.174	0.002	0.032
	**KEGG NAME**	**NES**	**p-value**	**FDR**
Positive	reactome_cell_cycle_checkpoints	2.705	0.002	0.012
	reactome_m_phase	2.620	0.001	0.012
	wp_retinoblastoma_gene_in_cancer	2.615	0.002	0.012
	reactome_DNA_replication	2.565	0.002	0.012
	reactome_mitotic_metaphase_and_anaphase	2.564	0.002	0.012
Negative	reactome_surfactant_metabolism	-2.051	0.002	0.013
	reactome_diseases_associated_with_surfactant_metabolism	-1.957	0.002	0.012
	asthma	-1.869	0.002	0.013
	linoleic_acid_metabolism	-1.854	0.002	0.013
	reactome_erythrocytes_take_up_carbon_dioxide_and_release_oxygen	-1.835	0.002	0.013

### Correlation Between *CDC25C* Expression and Immune Cell Infiltration

**Figure 5:**
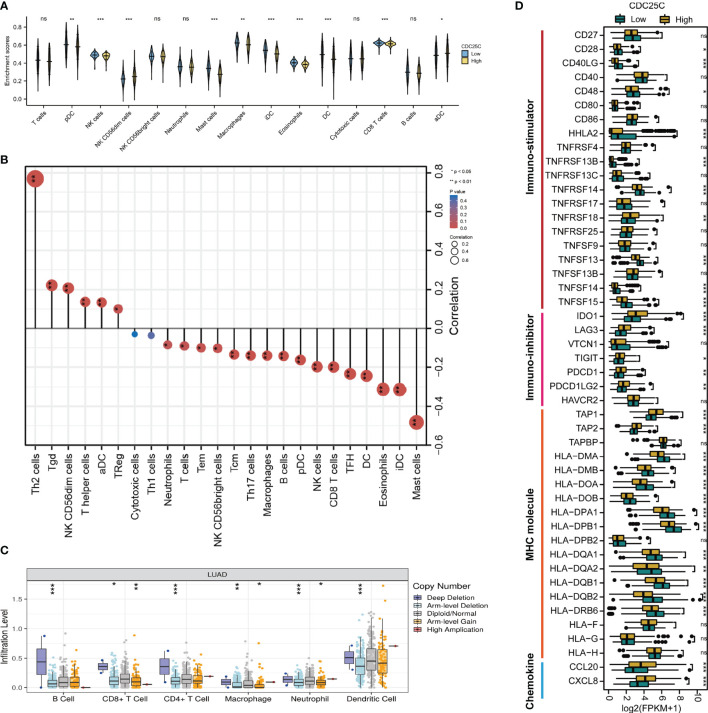
Characteristics of *CDC25C* immune cell infiltration. **(A)** Infiltration levels of various types of immune cells in high *CDC25C* and low *CDC25C* expression groups. **(B)** The correlation between *CDC25C* expression and immune cell infiltration. **(C)** Relationship between *CDC25C* copy number and immune cell infiltration in LUAD. **(D)** Comparison of the expression of immune-related signatures, including immuno-stimulators, immuno-inhibitors, MHC molecules, and chemokines, between high *CDC25C* and low *CDC25C* expression LUAD (not, not significant, *P < 0.05, **P < 0.01, ***P < 0.001).

The relationship between *CDC25C* expression and immune cells infiltration in the TME of LUAD tissues was further investigated. **Figure 5A** showed the infiltration of 15 immune cells in high- and low-*CDC25C* expression groups. Among these, the infiltration levels of 8 types of immune cells were lower in the high *CDC25C* expression group than in the low expression group, including CD8^+^T cells, pDC, iDC, DC, NK cells, mast cells, macrophages, and eosinophils (all P values < 0.05). In contrast, NK CD56dim cells and aDC had higher infiltration levels in the high *CDC25C* expression group (all P values < 0.05). Moreover, the correlation between *CDC25C* expression and infiltration of 24 immune cells was analyzed. Most immune cell infiltration negatively correlated with *CDC25C* expression, including CD8^+^ T cells, B cells, NK cells, and DC (all P values < 0.05) (**Figure 5B**). In contrast, *CDC25C* positively correlated with Th2 cells and Treg (all P values < 0.05) (**Figure 5B**). The TISIDB database was further explored, and similar results were obtained **(** **Figure S2** **)**. Moreover, it was observed that *CDC25C* copy number was related to immune cell infiltration in LUAD tissues. Deep deletion of *CDC25C* correlated with higher immune cell infiltration (**Figure 5C**).

Multiple immune-related signatures, including immuno-stimulators, immuno-inhibitors, MHC molecules, and chemokines were compared between high- and low- *CDC25C* expression LUAD. Regarding immuno-stimulators, it was demonstrated that *CD28*, *CD40LG*, *CD48*, *HHLA2*, *TNFRSF13B*, *TNFRSF14*, *TNFSF13*, *TNFSF14*, and *TNFSF15* were dramatically abundant in low-*CDC25C* expression LUAD (all P values < 0.05) (**Figure 5D**). Conversely, various immuno-inhibitors, including *IDO1*, *LAG3*, *TIGIT*, *PDCD1*, and *PDCD1LG2*, were more abundant in high-*CDC25C* expression LUAD (all P values < 0.05) (**Figure 5D**). In addition, we found that most MHC molecules were more highly expressed in low-*CDC25C* LUAD (most P values < 0.05) (**Figure 5D**). Besides, compared to that in low-*CDC25C* expression LUAD, chemokines, *CCL20* and *CXCL8*, were significantly higher in high-*CDC25C* expression LUAD (all P values < 0.05) (**Figure 5D**).

### Correlation Between *CDC25C* Expression and the Efficacy of Nivolumab in Patients With LUAD

**Figure 6:**
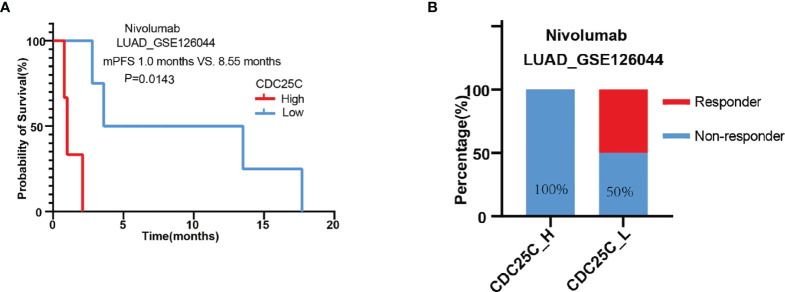
Correlation between *CDC25C* expression and outcomes of LUAD patients treated with nivolumab. **(A)** The correlation between *CDC25C* expression and PFS in LUAD patients treated with nivolumab. **(B)** The response rate between high and low *CDC25C* expression in LUAD patients treated with nivolumab.

To further verify the relationship between *CDC25C* expression and the efficacy of ICIs therapy, the GSE126044 dataset was downloaded, involving 16 patients receiving nivolumab, of which 7 patients with LUAD and 9 patients with LUSC. Firstly, the association of *CDC25C* expression with survival and response was analyzed using data from these 16 patients. There was a trend towards shorter PFS for non-small cell lung cancer patients with higher *CDC25C* expression (high vs. low: 1.55 months vs. 5.45 months, P = 0.0693) (**Figure S3A**). Besides, the non-response rate of 87.5% for patients with high *CDC25C* expression was higher than 50% for patients with low *CDC25C* expression (**Figure S3B**). Next, data regarding 7 LUAD samples were collected to further analyze the effect of *CDC25C* on the survival of LUAD patients treated with nivolumab. Notably, the analysis demonstrated that higher *CDC25C* expression was associated with shorter PFS (high vs. low: 1.0 month vs. 8.55 months, P = 0.0143) (**Figure 6A**). In addition, higher *CDC25C* expression correlated with lower response to nivolumab, with a response rate of 0% in the high *CDC25C* expression group and 50% in the low *CDC25C* expression group (**Figure 6B**).

### Identification of 10 Hub Genes Co-Expressed With *CDC25C* Using the STRING Database

**Figure 7:**
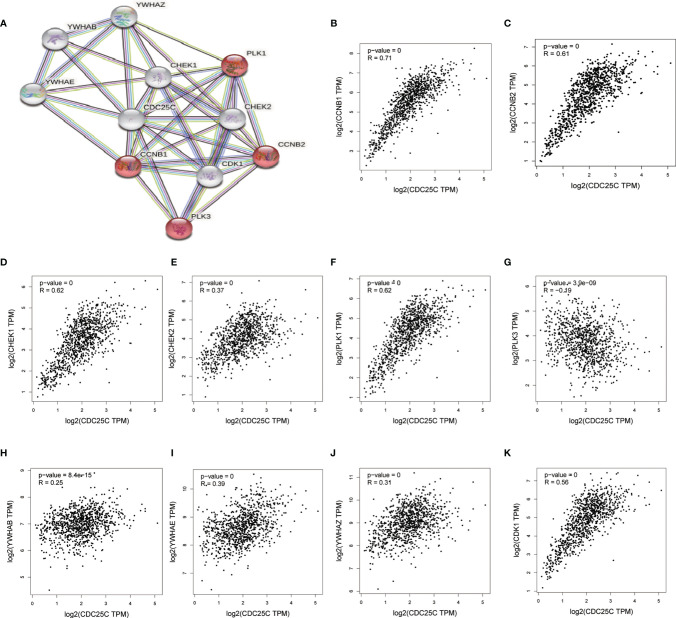
Identification of 10 hub genes closely related to *CDC25C* using STRING. **(A)** The PPI network plot for *CDC25C* including 10 hub genes co-expressed with *CDC25C* **(B–K)** Correlation analysis between *CDC25C* and 10 genes co-expressed with *CDC25C* using GEPIA (*CCNB1*, *CCNB2*, *CHEK1*, *CHEK2*, *PLK1*, *PLK3*, *YWHAB*, *YWHAE*, *YWHAZ*, *CDK1*).

**Figure 8:**
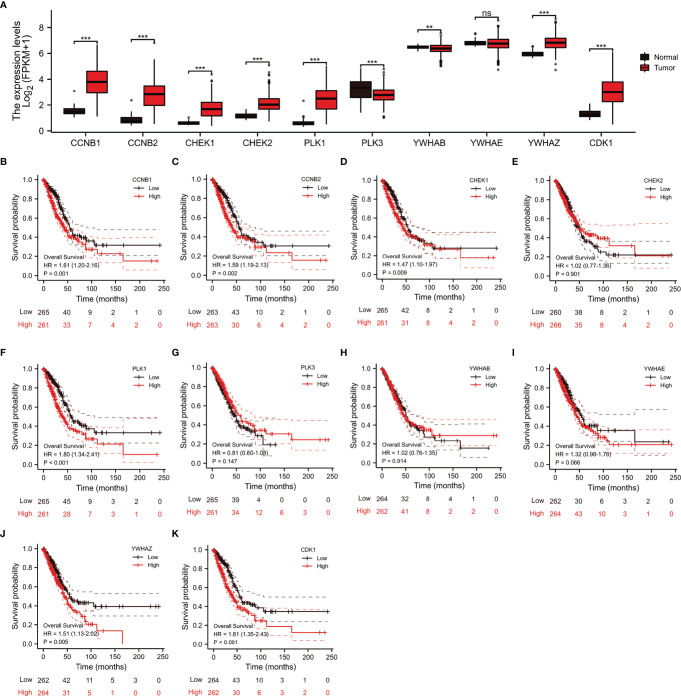
Analysis of the expression and prognostic significance of 10 genes in LUAD patients from the TCGA database. **(A)** Expression of 10 genes in LUAD versus normal tissue. **(B–K)** Association between 10 genes expression and OS in patients with LUAD (ns, not significant, **P < 0.01, ***P < 0.001).

Through the STRING database, we found 10 hub genes that were co-expressed with *CDC25C*, namely *CCNB1*, *CCNB2*, *CHEK1*, *CHEK2*, *PLK1*, *PLK3*, *YWHAB*, *YWHAE*, *YWHAZ* and *CDK1* (**Figure 7A**). By reviewing the literature, the main functions of these hub genes were summarized in **Table S1**. As shown in **Figures 7B–K**, these hub genes were closely related to *CDC25C*. Next, the expression of the 10 hub genes was explored using LUAD data from the TCGA database. The results showed that nine genes were differentially expressed in LUAD versus normal tissues (all P values < 0.01), in which seven genes were upregulated (*CCNB1*, *CCNB2*, *CHEK1*, *CHEK2*, *PLK1*, *YWHAZ* and *CDK1*) and two genes were downregulated (*PLK3* and *YWHAB*) (**Figure 8A**). Moreover, the same data was used to explore the prognostic value of these hub genes in LUAD, of which higher expression of six genes (*CCNB1*, *CCNB2*, *CHEK1*, *PLK1*, *YWHAZ* and *CDK1*) was associated with a shorter OS, while the other four genes (*CHEK2*, *PLK3*, *YWHAB* and *YWHAE*) were not significantly associated with OS (**Figures 8B** **–K**). Notably, the six co-expressed genes (*CCNB1*, *CCNB2*, *CHEK1*, *PLK1*, *YWHAZ* and *CDK1*) could compose a better predictive model for the survival of LUAD patients (**Figure S4**).

### The Expression and Prognostic Value of *CDC25C* in Pan-Cancer

**Figure 9:**
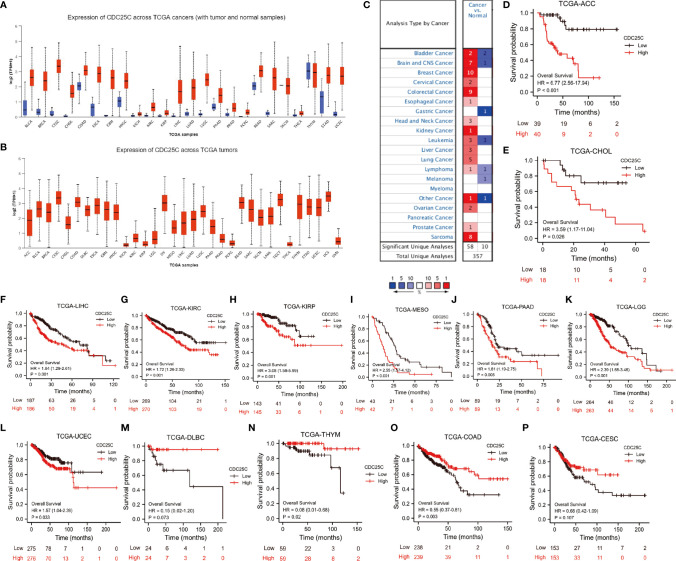
The Expression and prognostic value of *CDC25C* in Pan-Cancer. **(A)** Expression of *CDC25C* among multiple human tumors and corresponding normal tissues in the UALCAN database. ◼ is tumor, ◼ is normal tissue**. (B)** Expression of *CDC25C* in 33 tumors based on UALCAN database. **(C)** Expression of *CDC25C* among multiple human tumors and corresponding normal tissues in the Oncomine database. **(D–L)** In 9 kinds of tumors from the TCGA database, high *CDC25C* expression correlated with poor prognosis. **(M–P)** In 4 kinds of tumors from the TCGA database, higher *CDC25C* expression correlated with better prognosis. (ACC, adrenocortical carcinoma; CHOL, cholangiocarcinoma; LIHC, liver hepatocellular carcinoma; KIRC, kidney renal clear cell carcinoma; KIRP, kidney renal papillary cell carcinoma; MESO, kesothelioma; PAAD, pancreatic adenocarcinoma; LGG,brain lower grade glioma; UCEC, uterine corpus endometrial carcinoma; DLBC, lymphoid neoplasm diffuse large B-cell lymphoma; THYM, thymoma; COAD, colon adenocarcinoma; CESC, cervical squamous cell carcinoma and endocervical adenocarcinoma).

The pan-cancer analysis was performed to explore the role of *CDC25C* in other cancers. Through UALCAN database, the *CDC25C* expression was found significantly higher in 22 cancers when compared to that of normal tissues (**Figure 9A**). Similar results were obtained in the Oncomine database (**Figure 9C**). **Figure 9B** showed the relative amount of *CDC25C* expression in 33 cancers, in which *CDC25C* expression in LUAD was at an intermediate level. Strikingly, *CDC25C* expression was significantly correlated with the OS of 13 cancers, among which negative correlations were observed in 9 cancers (all P values < 0.05) (**Figures 9D** **–L**), and positive correlations were observed in 4 cancers (all P values < 0.05) (**Figures 9M** **–P**). Survival analysis in the other 18 cancers showed no evident correlation between *CDC25C* expression and OS (**Figure S5**). Based on the above analyses, it was revealed that the effect of *CDC25C* varied among different cancers.

## Discussion

The approval of ICIs in lung cancer is a significant milestone in the history of lung cancer treatment. However, only limited patients respond to immunotherapy. Even though predictive markers such as PD-L1 and TMB have been identified, clinicians failed to precisely and sensitively screen the population that would benefit from ICIs therapy (12). Therefore, more biomarkers are needed to accurately predict the efficacy of ICIs and provide clues to overcome drug resistance.

Previous studies have found that an abnormal cell cycle is closely related to uncontrollable tumor cell proliferation (37). *CDC25C*, a cell cycle regulatory protein, has been confirmed to be associated with tumorigenesis and tumor progression (38, 39). Consistently, LUAD of high *CDC25C* expression was mainly enriched in mitosis-related phases, indicating the critical role of *CDC25C* in LUAD proliferation. In the study, the expression of *CDC25C* and its relationship with the prognosis of LUAD patients were evaluated. *CDC25C* expression was upregulated in LUAD compared to normal tissue in multiple databases, including TCGA, Oncomine, and HPA. Furthermore, q-PCR showed that *CDC25C* expression was higher in LUAD cell lines (PC9 and H1299) than in the normal lung epithelial tissue cell line (BEAS-2B), which was consistent with the previously published report (40). In vulvar squamous cell carcinomas, overexpression of *CDC25C* was associated with a later FIGO stage (41). However, there are few studies on the association of *CDC25C* with LUAD. Our study revealed that higher *CDC25C* expression was associated with a shorter OS using multiple databases, including the TCGA and GEO databases. Moreover, multivariate and univariate Cox regression analyses confirmed that *CDC25C* was an independent prognostic factor in LUAD. In addition, higher *CDC25C* expression correlated with a later T-stage, N-stage, M-stage, and pathological stage, suggesting that *CDC25C* could be a potential biomarker for tumor staging. *TP53*, a tumor suppressor gene regulating cell cycle transcription (42), whose mutation is associated with an unrestricted cell cycle (43). In line with this, higher *CDC25C* expression was observed in *TP53* mutant tumors, suggesting that *CDC25C* may be an essential mediator of *TP53*-mediated tumor progression.

Multiple studies have proven that abnormal cell cycle activity is strongly associated with chemotherapy resistance (17). Notably, recent studies have revealed that cell cycle-related genes could modulate the TME influencing the efficacy of immunotherapy (44). *CDK4/6* inhibitors have been proved to promote CD8^+^ T memory cell formation, thereby enhancing anti-tumor immunity (26). Besides, *CDK7* inhibitors could enhance the efficacy of anti-PD-1 therapy in NSCLC (27, 45). Additionally, Petroni etc. have revealed that the amplification of *cyclin D1* and *CDK4*, two cell cycle-related genes, was associated with a low response to ICIs treatment in solid tumors (46). Strikingly, our study first demonstrated that higher *CDC25C* expression was associated with shorter PFS and lower response rate in LUAD patients treated with nivolumab, suggesting that *CDC25C* is likely to be associated with immunotherapy efficacy. Given that the analysis was based on 7 LUAD patients, its reliability extremely requires further validation with larger samples. It is acknowledged that TME is a critical factor in modulating the efficacy of immunotherapy (47). In our study, CDC25C was significantly associated with the infiltration levels of multiple immune cells, with negative correlations with anti-tumor immune cells (CD8+ T cells, B cells, NK cells, and DC) (48), but positive correlations with immunosuppressive immune cells (Th2 and Treg cells) (48). In other words, these findings indicated that *CDC25C* promotes an immunosuppressive TME, which could impair the efficacy of immunotherapy. Additionally, immuno-stimulators and immuno-inhibitors are crucial in modulating cancer-immunity cycle (49). Higher levels of immuno-stimulators and lower levels of immuno-inhibitors in low *CDC25C* LUAD could promote enhanced anti-tumor immunity, supporting prolonged survival of patients with low *CDC25C*. MHC molecules, playing an essential role in antigen presentation, are indispensable in the recognizing and killing of tumor cells by immune cells (50). Lower MHC molecules in high *CDC25C* LUAD could mediate tumor immune escape, weakening the efficacy of immunotherapy. Higher *CCL20* and *CXCL8* in high *CDC25C* LUAD, which could attract Treg cells into tumors to form an immunosuppressive TME (51), providing stronger evidence for poor prognosis in nivolumab-treated patients with high *CDC25C*. Taken together, these findings suggested that high *CDC25C* facilitates an immunosuppressive TME, providing potential mechanisms for shortened survival and low response in nivolumab-treated patients with high *CDC25C* and potentially paving the way for the combination of *CDC25C* inhibitors with immunotherapy. Growing evidence suggests the critical role of metabolism in immunotherapy (52). Strong correlation of *CDC25C* with metabolism-related processes suggested that metabolism may be another vital mechanism underlying the impact of *CDC25C* on immunotherapy.

The expression and prognostic value of *CDC25C* in 31 tumors were further explored. Of note, prolonged survival with high *CDC25C* expression was observed in several cancers. Indeed, the effects of genes on cancer progression or prognosis are not only associated with intrinsic properties of genes themselves, but also subject to a variety of factors, such as treatment regimens and tumor types (53). Although there is sufficient evidence that *CDC25C* promotes tumor progression, high *CDC25C* expression enhances the sensitivity of esophageal cancer to radiotherapy (54). *KRAS*, the oncogene mutated highly in cancers, is associated with poor prognosis of LUAD (55). However, patients harboring *KRAS* mutation are more vulnerable to benefit from ICIs (56). In addition, *ID4* acts as a tumor suppressor in prostate cancer, while serves as a proto-oncogene in bladder cancer (57). Therefore, we hypothesize that the disparity of prognosis for *CDC25C* in diverse tumors may also be related to these factors.

Here we demonstrated that a higher *CDC25C* expression was associated with shorter survival and lower response to ICIs, as well as an immunosuppressive tumor microenvironment, in patients with LUAD. These finding suggest that targeting inhibition of key regulators of cell cycle in cancers might augment anti-tumor immunity and increase the response to ICIs treatment, which provide a rational for further combination therapies. Even though a comprehensive and systematic analysis was performed, there are still some limitations. The data in this study were extracted mainly from public and different databases, and some specific information is not available, so our results are prone to errors. Although we first confirmed the role of *CDC25C* in ICIs treatment, the conclusion is likely to be biased due to the small sample size, thus validation in a larger sample is extremely warranted. Besides, the potential mechanism of how *CDC25C* modulates the efficacy of immunotherapy requires more investigation using *in vitro* experiments and *in vivo* animal models. Nevertheless, multiple methods were used to validate and similar results were obtained in our analyses, giving a more reliable relationship that high *CDC25C* expression correlated with an immunosuppressive TME of LUAD. Undeniably, since the study focused on the relationship between *CDC25C* and LUAD, only survival and expression analyses were performed in the pan-cancer. More in-depth studies of each type of cancer are warranted.

## Conclusion

In conclusion, it was demonstrated that *CDC25C* expression was upregulated in LUAD compared to normal lung tissue at the mRNA and protein levels. Based on survival analysis, *CDC25C* was confirmed to be an independent risk factor for LUAD. Most importantly, higher *CDC25C* expression was associated with a shorter PFS and lower response rate for LUAD patients treated with nivolumab, which provided more robust evidence for the role of the cell cycle in immunotherapy. In addition, higher *CDC25C* expression was associated with an immunosuppressive TME, suggesting the role of cell cycle in the TME modulation.

## Data Availability Statement

The datasets presented in this study can be found in online repositories. The names of the repository/repositories and accession number(s) can be found in the article/**Supplementary Material**.

## Author Contributions

XW and YL conceived and guide the study. WZ and XS collected the data, performed analysis, and drafted the manuscript. FY, NL, WH, and HX collected the literature, edited figures and revised the manuscript. All authors contributed to the article and approved the final version.

## Funding

This work was supported by grants from the National Natural Science Foundation of China (No. 81874044) and the Shandong Provincial Natural Science Foundation (No.ZR2020MH236 and No. ZR2019MH050).

## Conflict of Interest

The authors declare that the research was conducted in the absence of any commercial or financial relationships that could be construed as a potential conflict of interest.

## Publisher’s Note

All claims expressed in this article are solely those of the authors and do not necessarily represent those of their affiliated organizations, or those of the publisher, the editors and the reviewers. Any product that may be evaluated in this article, or claim that may be made by its manufacturer, is not guaranteed or endorsed by the publisher.

## Abbreviations

*CDC25C*, cell division cycle 25c; DSS, disease specific survival; GEO, Gene Expression Omnibus; GO, Gene Ontology; GSEA, Gene Set Enrichment Analysis; HPA, The Human Protein Atlas; ICIs, immune checkpoint inhibitors; KEGG, Kyoto Encyclopedia of Genes and Genomes; LUAD, lung adenocarcinoma; LUSC, lung squamous carcinoma; NSCLC, non-small cell lung cancer; OS, overall survival; q-PCR, real-time quantitative polymerase chain reaction; PFS, progression free survival; PFI, progress free interval; PPI, protein-protein interaction; TCGA, The Cancer Genome Atlas; TMB, tumor mutational burden; TME, tumor microenvironment.
