# Supplementary material for: CDC25C as a Predictive Biomarker for Immune Checkpoint Inhibitors in Patients With Lung Adenocarcinoma
Source: Front Oncol. 2022 Apr 29;12:867788. doi: 10.3389/fonc.2022.867788 (PMC9104567; doi:10.3389/fonc.2022.867788)
Supplement: Supplementary file 1 [file DataSheet_1.zip › Supplementary figures.docx]

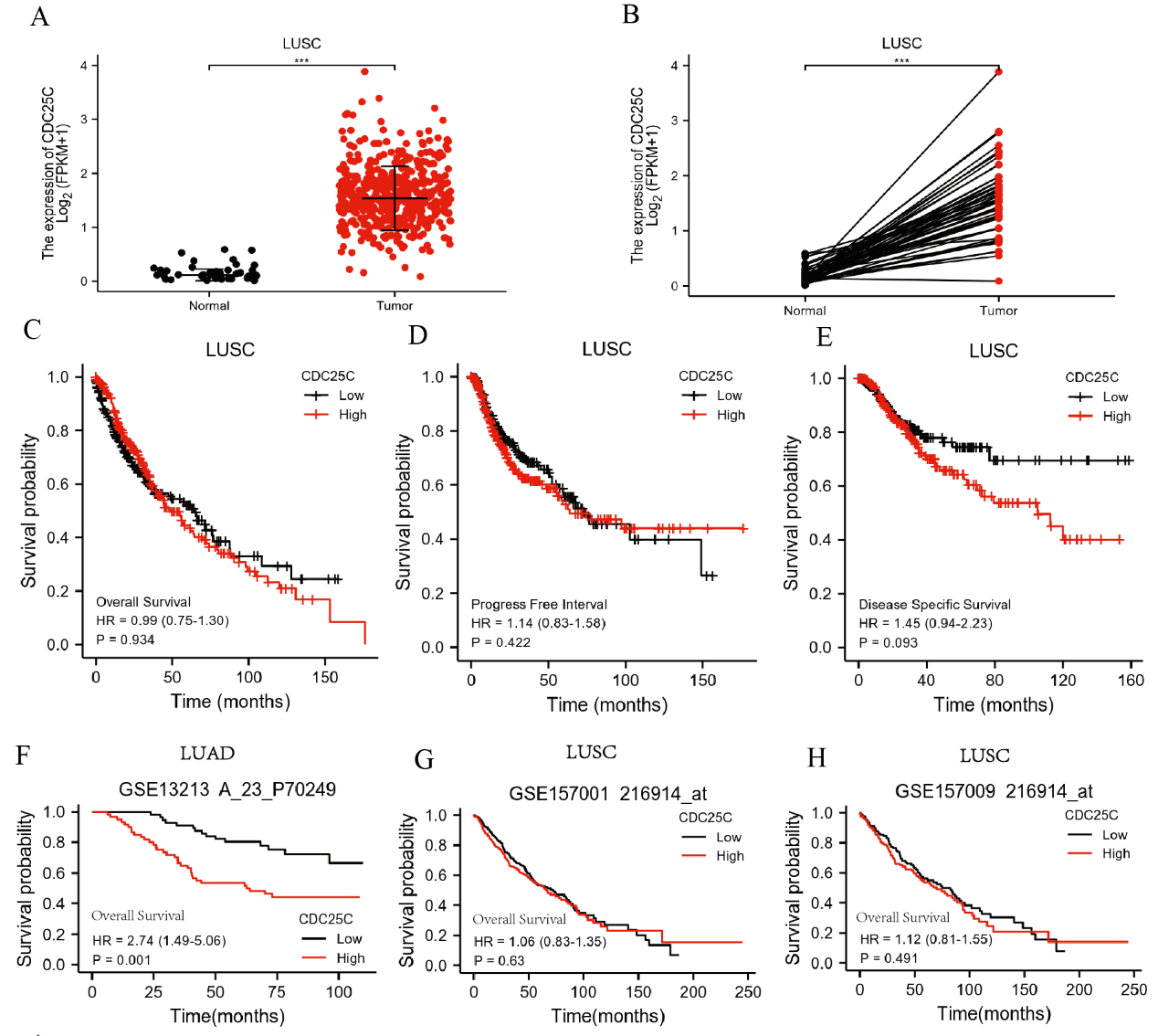


**FIUGURE S1| (A-B)** *CDC25C* expression between tumors and normal tissues of non-paired (**A**) and paired(**B**) LUSC patients from the TCGA database. **(C-E)** Correlation among *CDC25C* expression and OS (**C**), PFI (**D**), DSS (**E**) of LUSC in TCGA database. **(F)** Correlation between *CDC25C* expression and OS of LUAD in GEO database. **(G-H)** Correlation between *CDC25C* expression and OS of LUSC in GEO database.


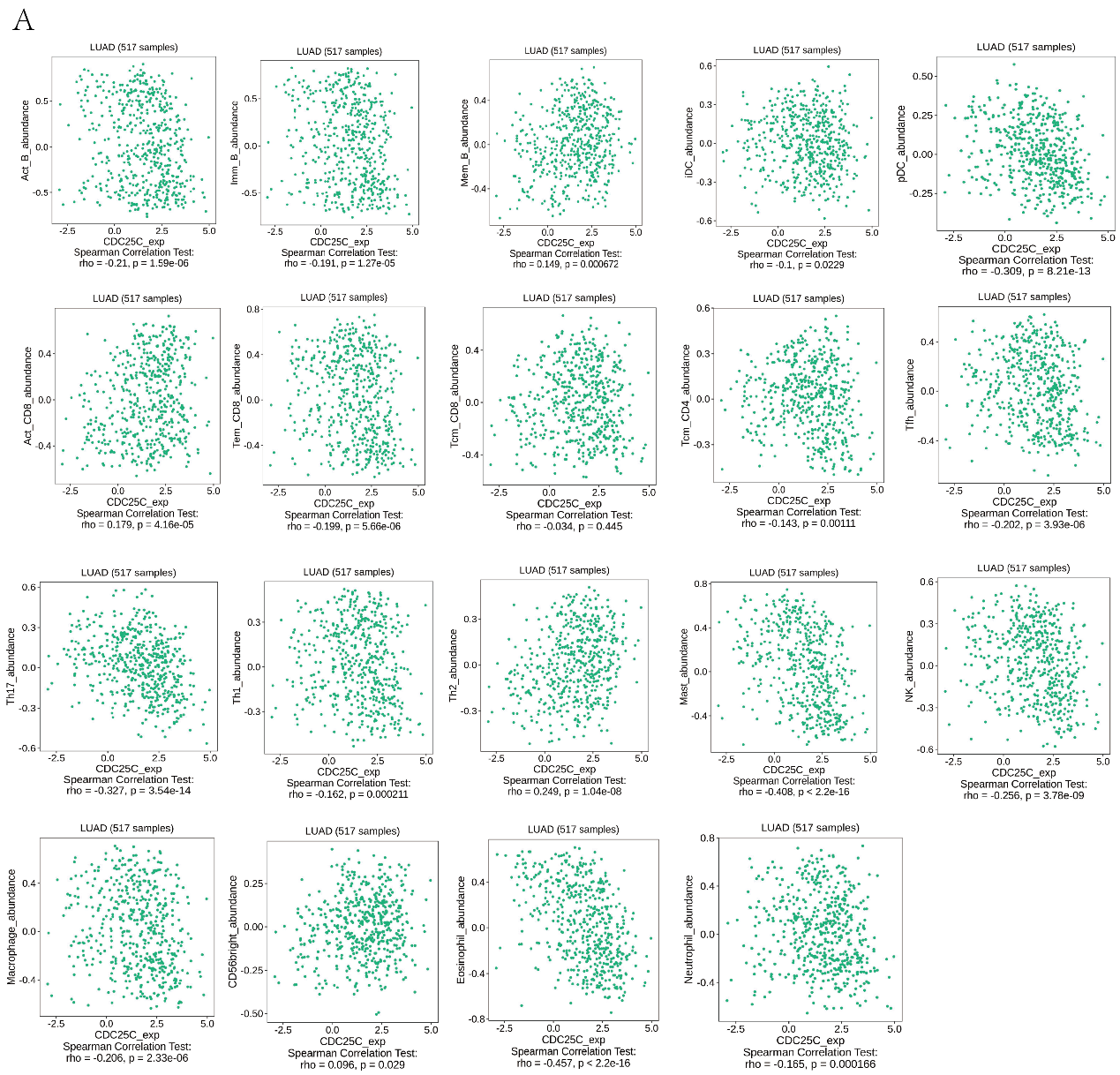


**FIGURE S2 |** **(A)** Relationship between *CDC25C* expression and immune cell infiltration of LUAD in the TISIDB database (Act_B: activated CD8 T cell; Imm_B: immature B cell; Mem_B: memory B cell; iDC: immature dendritic cell; pDC: plasmacytoid dendritic cell; Act_CD8: activated CD8 T cell; Tem_CD8: effector memory CD8 T cell; Tcm_CD8: central memory CD8 T cell; Tcm_CD4: central memory CD4 T cell; Tfh: T follicular helper cell; Th17: type 17 T help cell; Th1: type 1 T help cell; Th2: type 2 T help cell; Mast: mast cell; NK: natural killer T cell; CD56bright: CD56 bright natural killer cell).


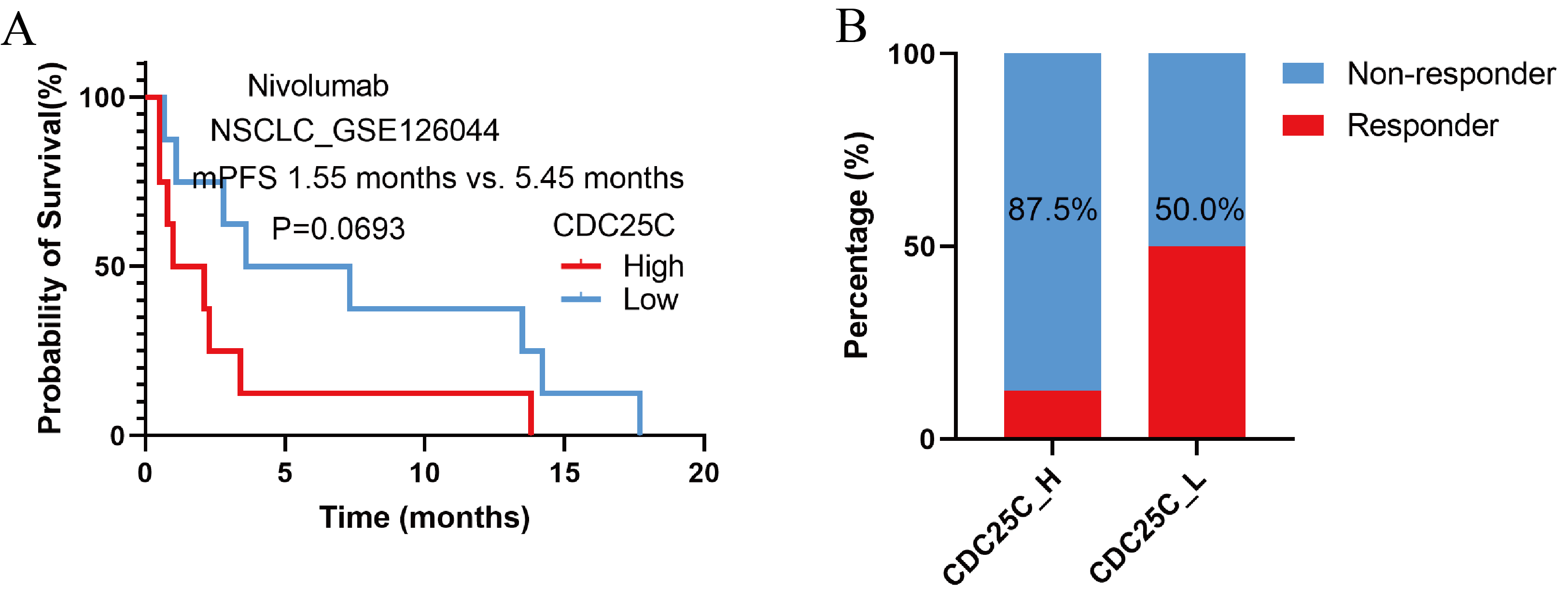


**FIGURE S3 |** Correlation between *CDC25C* expression and outcomes of NSCLC patients treated with nivolumab. (**A**) The correlation between *CDC25C* expression and PFS in NSCLC patients treated with nivolumab. (**B**) The response rate between high and low *CDC25C* expression in NSCLC patients treated with nivolumab.


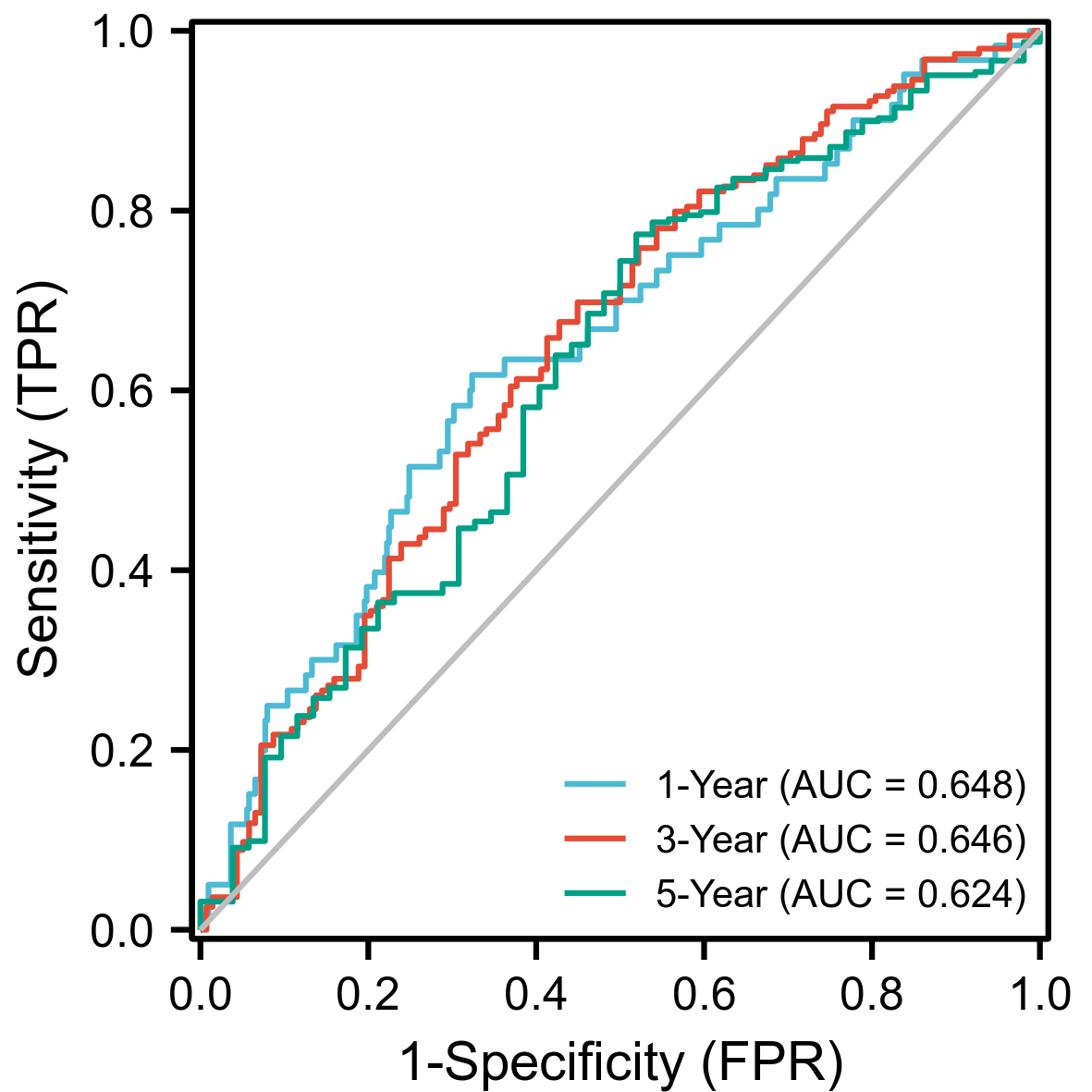


**FIGURE S4**| The AUC curve of 1, 3 and 5 years to forecast the survival of LUAD patients with six co-expressed genes (*CCNB1*, *CCNB2*, *CHEK1*, *PLK1*, *YWHAZ* and *CDK1*).


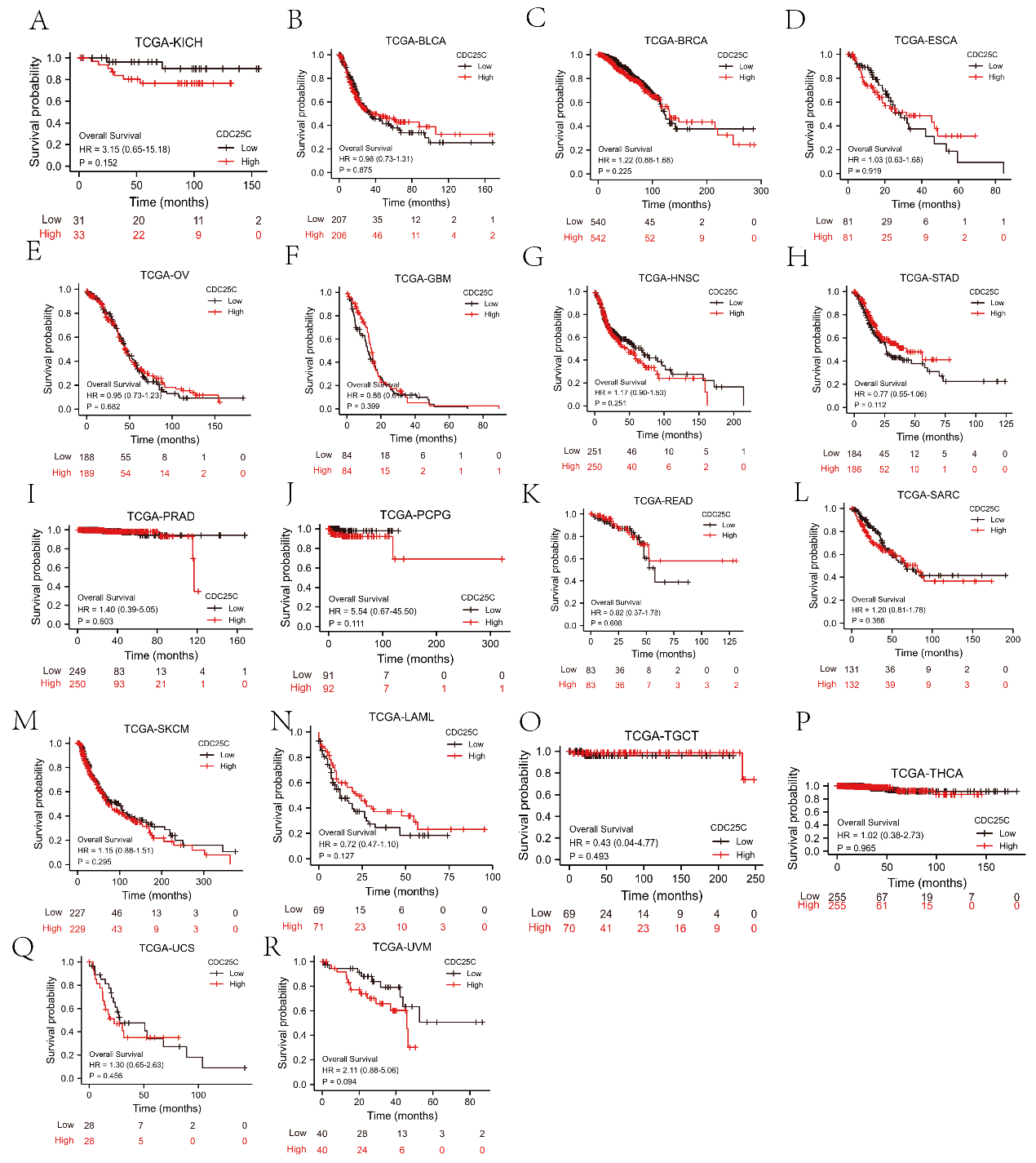


**FIGURE S5 |** The prognostic value of *CDC25C* in 18 tumors. **(A-R)** In these 18 tumors, analysis showed no correlation between *CDC25C* expression and OS (KICH: Kidney Chromophobe, BLCA: Bladder Urothelial Carcinoma, BRCA: Breast invasive carcinoma, ESCA: Esophageal carcinoma, OV: Ovarian serous cystadenocarcinoma, GBM: Glioblastoma multiforme, HNSC: Head and Neck squamous cell carcinoma, STAD: Stomach adenocarcinoma, PRAD: Prostate adenocarcinoma, PCPG: Pheochromocytoma and Paraganglioma, READ: Rectum adenocarcinoma, SARC: Sarcoma, SKCM: Skin Cutaneous Melanoma, LAML: Acute Myeloid Leukemia, TGCT: Testicular Germ Cell Tumors, THCA: Thyroid carcinoma, UCS: Uterine Carcinosarcoma, UVM: Uveal Melanoma).
