# Supplementary material for: CDC25C as a Predictive Biomarker for Immune Checkpoint Inhibitors in Patients With Lung Adenocarcinoma
Source: Front Oncol. 2022 Apr 29;12:867788. doi: 10.3389/fonc.2022.867788 (PMC9104567; doi:10.3389/fonc.2022.867788)
Supplement: Supplementary file 1 [file DataSheet_1.zip › Supplementary table.docx]

**Table S1. Functional roles of 10 hub genes.**

| NO. | Gene symbol | Full name | Summary |
| --- | --- | --- | --- |
| 1 | *CHEK1* | checkpoint kinase 1 | *CHEK1*, also known as *CHK1,* a member of the *CHEK* family, the protein encoded by this gene belongs to the Ser/Thr protein kinase family. *CHK1*  has been shown to act as essential roles in DNA replication, mitotic progression, DNA repair, and overall cell cycle regulation. Additionally, it was shown that *CHEK1* was highly expressed in a variety of solid tumors and is associated with poor prognosis in multiple tumors (1) |
| 2 | *CHEK2* | checkpoint kinase 2 | *CHEK2*, so-called *CHK2*, is a versatile kinase that is responsible for regulating the cellular response to DNA damage, cell-cycle arrest, and activation of apoptosis. It was demonstrated that *CHEK1* acts as a tumor suppressor, but differs from a classical tumor suppressor gene (2). |
| 3 | *CCNB1* | cyclin B1 | *CCNB1*, forming a complex with *CDK1*, promote the transition from the G2 phase of cell cycle to mitosis. There is emerging evidence that *CCNB1* is associated with tumorigenesis and is highly expressed in human tumors (3). |
| 4 | *CCNB2* | cyclin B2 | *CCNB2*, a member of the cyclin family, serves a pivotal role in the G2/M transition. It has been shown to regulate the activity of cell cycle and different cyclins function. *CCNB1* expression is upregulated in human cancers and is associated with poor prognosis (4). |
| 5 | *PLK1* | polo-like kinase 1 | *PLK1*, belonging to the polo sub-family of Ser/Thr protein kinases, which is strongly correlated with cell cycle progression, differentiation, and survival. *PLK1* is overexpressed in several types of cancer cells, resulting in genome instability and enabling tumorigenesis. *PLK1* also mediates tumor resistance to chemotherapy and radiotherapy (5). |
| 6 | *PLK3* | polo-like kinase 3 | *PLK3*, belonging to the polo sub-family of Ser/Thr protein kinases, acts as a key gene in regulation of cell cycle progression and tumorigenesis (6). |
| 7 | *YWHAE* | Tyrosine3-monooxygenase /tryptophan5-monooxygenase activation protein, epsilon | *YWHAE*, belonging to the 14-3-3 protein family, which modulates signal transduction through the phosphorylation of serine/threonine motifs of target proteins. *YWHAE* interacts with *CDC25* phosphatases and correlates with cell division. Accumulating evidence indicates that the *PLK3* plays a critical role in cancers (7). |
| 8 | *YWHAB* | tyrosine3-monooxygenase/tryptophan5-monooxygenase activation protein, beta | *YWHAB*, belonging to the 14-3-3 protein family, has been shown to be associated with lung cancer properties (8). |
| 9 | *YWHAZ* | tyrosine3-monooxygenase/ tryptophan5-monooxygenase activation protein, zeta | *YWHAZ* (also named 14-3-3ζ), belonging to the 14-3-3 protein family, is a central hub protein involved in many signal transduction pathways and plays a key role in many tumor activities, including cell growth, cell cycle, apoptosis, invasion (9). |
| 10 | *CDK1* | cyclin-dependent kinase 1 | *CDK1*, a member of the Ser/Thr protein kinase family, plays a important regulatory role in cell cycle control. In addition, it was revealed that *cyclin B1/CDK1*-regulated mitochondrial activity was associated with tumor proliferation and drug resistance. Alternatively, *CDK1* was found to be a new target for the treatment of pancreatic ductal adenocarcinoma (10, 11) |

#### Reference

1. Neizer-Ashun F, Bhattacharya R. Reality CHEK: Understanding the biology and clinical potential of CHK1. *Cancer Lett* (2021): 202-211.doi: 10.1016/j.canlet.2020.09.016

2. Antoni L, Sodha N, Collins I, Garrett MD. CHK2 kinase: cancer susceptibility and cancer therapy - two sides of the same coin? *Nat Rev Cancer* (2007) 12: 925-936.doi: 10.1038/nrc2251

3. Fang Y, Yu H, Liang X, Xu J, Cai X. Chk1-induced CCNB1 overexpression promotes cell proliferation and tumor growth in human colorectal cancer. *Cancer Biol Ther* (2014) 9: 1268-1279.doi: 10.4161/cbt.29691

4. Qian X, Song X, He Y, Yang Z, Sun T, Wang J et al. CCNB2 overexpression is a poor prognostic biomarker in Chinese NSCLC patients. *Biomed Pharmacother* (2015): 222-227.doi: 10.1016/j.biopha.2015.08.004

5. Iliaki S, Beyaert R, Afonina IS. Polo-like kinase 1 (PLK1) signaling in cancer and beyond. *Biochem Pharmacol* (2021): 114747.doi: 10.1016/j.bcp.2021.114747

6. Helmke C, Becker S, Strebhardt K. The role of Plk3 in oncogenesis. *Oncogene* (2016) 2: 135-147.doi: 10.1038/onc.2015.105

7. Yang YF, Lee YC, Wang YY, Wang CH, Hou MF, Yuan SF. YWHAE promotes proliferation, metastasis, and chemoresistance in breast cancer cells. *Kaohsiung J Med Sci* (2019) 7: 408-416.doi: 10.1002/kjm2.12075

8. Xu C, Du Z, Ren S, Liang X, Li H. MiR-129-5p sensitization of lung cancer cells to etoposide-induced apoptosis by reducing YWHAB. *J Cancer* (2020) 4: 858-866.doi: 10.7150/jca.35410

9. Gan Y, Ye F, He XX. The role of YWHAZ in cancer: A maze of opportunities and challenges. *J Cancer* (2020) 8: 2252-2264.doi: 10.7150/jca.41316

10. Xie B, Wang S, Jiang N, Li JJ. Cyclin B1/CDK1-regulated mitochondrial bioenergetics in cell cycle progression and tumor resistance. *Cancer Lett* (2019): 56-66.doi: 10.1016/j.canlet.2018.11.019

11. Wijnen R, Pecoraro C, Carbone D, Fiuji H, Avan A, Peters GJ et al. Cyclin Dependent Kinase-1 (CDK-1) Inhibition as a Novel Therapeutic Strategy against Pancreatic Ductal Adenocarcinoma (PDAC). *Cancers (Basel)* (2021) 17.doi: 10.3390/cancers13174389
